# The (cost-)effectiveness of an implemented fall prevention intervention on falls and fall-related injuries among community-dwelling older adults with an increased risk of falls: protocol for the in balance randomized controlled trial

**DOI:** 10.1186/s12877-021-02334-3

**Published:** 2021-06-23

**Authors:** Maaike van Gameren, Daniël Bossen, Judith E. Bosmans, Bart Visser, Sanne W. T. Frazer, Mirjam Pijnappels

**Affiliations:** 1 Department of Human Movement Sciences, Faculty of Behavioural and Movement Sciences, Amsterdam Movement Sciences Research Institute, Vrije Universiteit Amsterdam, Amsterdam, Netherlands; 2 Faculty of Health, Centre of Expertise Urban Vitality, Amsterdam University of Applied Sciences, Amsterdam, Netherlands; 3 Department of Health Sciences, Faculty of Science, Amsterdam Public Health Research Institute, Vrije Universiteit Amsterdam, Amsterdam, Netherlands; 4 Consumer Safety Institute (VeiligheidNL), Amsterdam, Netherlands

**Keywords:** Fall prevention, Effectiveness, Ageing, Elderly, Accidental falls, Intervention studies, Cost-effectiveness, Healthcare utilization

## Abstract

**Background:**

Falls and fall-related injuries among older adults are a serious threat to the quality of life and result in high healthcare and societal costs. Despite evidence that falls can be prevented by fall prevention programmes, practical barriers may challenge the implementation of these programmes. In this study, we will investigate the effectiveness and cost-effectiveness of In Balance, a fourteen-week, low-cost group fall prevention intervention, that is widely implemented in community-dwelling older adults with an increased fall risk in the Netherlands. Moreover, we will be the first to include cost-effectiveness for this intervention. Based on previous evidence of the In Balance intervention in pre-frail older adults, we expect this intervention to be (cost-)effective after implementation-related adjustments on the target population and duration of the intervention.

**Methods:**

This study is a single-blinded, multicenter randomized controlled trial. The target sample will consist of 256 community-dwelling non-frail and pre-frail adults of 65 years or older with an increased risk of falls. The intervention group receives the In Balance intervention as it is currently widely implemented in Dutch healthcare, which includes an educational component and physical exercises. The physical exercises are based on Tai Chi principles and focus on balance and strength. The control group receives general written physical activity recommendations. Primary outcomes are the number of falls and fall-related injuries over 12 months follow-up. Secondary outcomes consist of physical performance measures, physical activity, confidence, health status, quality of life, process evaluation and societal costs. Mixed model analyses will be conducted for both primary and secondary outcomes and will be stratified for non-frail and pre-frail adults.

**Discussion:**

This trial will provide insight into the clinical and societal impact of an implemented Dutch fall prevention intervention and will have major benefits for older adults, society and health insurance companies. In addition, results of this study will inform healthcare professionals and policy makers about timely and (cost-)effective prevention of falls in older adults.

**Trial registration:**

Netherlands Trial Register: NL9248 (registered February 13, 2021).

## Background

Falls are a major cause of mortality and morbidity in older adults [1]. More than one-third of community-dwelling adults aged 65 years or older falls at least once per year [2]. The number of falls is expected to increase even further due to the ageing of the population [3–5]. Falls in older adults often result in injury with 109,000 older adults visiting an Emergency Department due to a fall-related injury in the Netherlands in 2019 [6]. Besides the risk of injuries, falls may also increase fear of falling [7]. Fear of falling may result in a reduction in physical activity in daily life, which can lead to a decreased muscle strength and an increased fall risk [8–11]. Falls and its sequelae can lead to a decreased quality of life up to 9 months after a fall, suggesting that even after that amount of time older adults are still suffering from their fall and its consequences [12, 13]. Fall-related injuries result in considerable healthcare costs, which are estimated at more than 1 billion euros in the Netherlands [6]. On top of these healthcare costs, other costs also have to be taken into account, such as individual expenses, help from family and/or friends and productivity losses due to absence from work or unpaid activities [14–16]. The alarming impact of falls on older individuals, healthcare and our greying society renders the prevention of falls and fall-related injuries highly urgent [17, 18].

Systematic reviews have shown fall prevention programmes to be effective in reducing the risk of falls among older adults [19, 20]. Group interventions appear more effective than individual programmes, because of higher compliance of participants in the group intervention [21, 22]. Despite the evidence that falls can be prevented by fall prevention programmes, barriers for practical implementation may harm the impact of these programmes. It can for example be questioned whether effectiveness maintains after adjustments in the intervention when implemented, which indicates the importance of re-evaluating implemented interventions [18–20].

An effective group fall prevention intervention, that is widely implemented in the Netherlands, is the In Balance intervention. In Balance is a relatively inexpensive fourteen-week group intervention that requires minimal equipment and aims to reduce falls by increasing awareness, balance and strength in older adults at risk of falling [23]. The first four weeks of In Balance include counselling and education meetings once per week on topics regarding fall prevention to increase knowledge about preventing falls, and to increase awareness of fall risk and balance disruption. The last ten weeks comprise two one-hour exercise sessions per week. Exercises are derived from principles of Tai Chi and are mainly focused on physical balance and strength.

In 2006, Faber and colleagues demonstrated that the 20-week In Balance intervention resulted in a fall-risk reduction of 61% in pre-frail older adults, compared to usual care in a residential care setting, whereas in frail older adults the risk of becoming a faller increased considerably [24]. This difference in effect may be because frail participants were not able to perform the exercises as effectively as the pre-frail group [25]. Based on the positive findings in the pre-frail group, the In Balance fall prevention programme has been widely implemented in the Netherlands by certified therapists. After implementation, the target group was extended to a group mainly consisting of independently living older adults. Moreover, the duration of the intervention was shortened from twenty to fourteen weeks, for practical reasons and adherence, based on indications that the largest physical improvements in the study by Faber and colleagues were seen in this initial period [24].

Considering the adjustments in both duration and target population of the original In Balance intervention, re-evaluation of the effectiveness of the intervention is urgently needed. Moreover, evidence on the cost-effectiveness of fall prevention programmes is scarce [26]. Cost-effectiveness analysis of the In Balance intervention would allow insurance companies, policy- and decision makers to allocate health resources efficiently. Therefore, this study aims to assess the effectiveness and cost-effectiveness from a societal perspective of the In Balance fall prevention intervention compared to written general physical activity recommendations on the number of falls with and without injuries for community-dwelling adults of 65 years or older with an increased fall risk, stratified for frailty status (non-frail and pre-frail).

## Methods

### Study design and setting

This study is a single-blinded, multicenter randomized controlled trial with stratification on frailty levels. The design of the trial follows the recommendations of the Standard Protocol Items: Recommendations for Interventional Trials 2013 Checklist [27]. The study intervention will be executed by trained, certified and registered physical- and exercise therapists in several municipalities in the Netherlands. Vrije Universiteit Amsterdam is the sponsor of this study.

### Participants

The target population consists of community-dwelling older adults (aged ≥65 years) with an increased risk of falls according to the fall risk screening questionnaire [28–30]. All participants should be able to independently execute activities of daily living (e.g., going to the bathroom, dressing and undressing) and walk 100 m. Older adults will be included if they are classified as non-frail or pre-frail based on the phenotype concept introduced by Fried et al. [31]. Table 1 lists the in- and exclusion criteria including the operationalization for each criterion. Potential participants will be excluded if they participated in a fall prevention intervention in the past 6 months, if they are unable to read and understand Dutch, if they have cognitive impairment defined as a score < 19 on the Mini-Mental State Examination [32], or if they have any self-reported uncontrolled comorbid conditions or contraindications for conducting physical exercises during the In Balance intervention (e.g., cardiovascular, neurological and orthopaedic problems).

**Table 1:** Inclusion and exclusion criteria

Inclusion criteria	Exclusion criteria
• Adults aged ≥65 years • Classified as non-frail or pre-frail based on the phenotype concept introduced by Fried et al. (weight loss, weak grip strength, exhaustion, slow gait speed and low physical activity) [31] • Living in a community-based setting • Having a potential fall risk, as assed by the fall risk screening questionnaire [28–30] • Able to independently execute activities of daily living and walk 100 m	• Cognition < 19 points on the Mini-Mental State Examination [32] • Not able to read or understand Dutch • Participation in a fall prevention programme in the past 6 months • Self-reported contra-indications for participation in the In Balance intervention

#### Frailty status

Frailty status will be determined at baseline based on the frailty indicators according to Fried and colleagues [31]. The frailty phenotype includes weight loss, weak grip strength, exhaustion, slow gait speed and low physical activity [31]. Weight loss is assessed by self-reported, unintentional weight loss of 5 kg or more in the last year [31]. Grip strength will be assessed using a handheld dynamometer and is calculated as the average of three measurements of the score in kilogram of the dominant hand [33]. Original cut-off points stratified by sex and body mass index will be used to indicate weak grip strength [31]. Exhaustion is considered present if a participant has a score < 75 on the vitality subscale of the 36-Item Short Form Health Survey [34]. Gait speed will be assessed by recording the time in seconds to walk 4 m at usual pace; slow gait is defined using predefined cut-off points, converted to a 4 m distance, stratified by sex and height [31]. Last, low physical activity will be assessed using the International Physical Activity Questionnaire Short Form (IPAQ-SF) and is defined as burning less than 383 or 270 kcal with physical activity per week for men and women, respectively [31, 35]. Participants will be considered as non-frail if they meet none of the criteria and classified as pre-frail when meeting 1 or 2 of the criteria. If participants meet 3 or more of the criteria, they will be classified as frail and will be excluded from this study [31].

### Recruitment, randomization, blinding and treatment allocation

Recruitment will be done via flyers, advertisements in local papers, folders distributed through supermarkets, pharmacists, community centres, general practitioners, at annual flu shots, and via personal invitations by In Balance therapists and general practitioners. Participants will be randomized into the intervention group or the control group using block-randomization to balance the size of the two groups. Randomization will be stratified according to frailty. We aim to include 30–50% of the participants in the pre-frail subgroup and 50–70% in the non-frail subgroup in both the intervention and control group. As soon as one of the subgroups starts to outnumber the other subgroup, we will only target the underrepresented subgroup for recruitment. Two hundred fifty-six sealed envelopes in blocks of 10 (5 envelopes per group) will be prepared by an independent investigator. These sequentially numbered envelopes contain a random computer-generated unique identification number. A second matching envelope contains the group information. All investigators and assessors involved in this study will be blinded to group assignment until completion of statistical analyses. Due to the nature of this study, participants and therapists cannot be blinded to group allocation. Participants will be informed orally about their allocation and therapists will receive the names of those participants who will participate in the In Balance intervention.

### Informed consent

When potential participants have shown interest to participate in the study by contacting one of the investigators, they will first be interviewed by telephone on their eligibility according to the in- and exclusion criteria. Eligible participants will receive an extended information letter via mail or email, in which the study procedures, including randomization, will be explained. All potential participants will be given at least one week to consider participation and to approach the investigator for questions. Eligible participants will be contacted by phone and asked whether they want to participate in the study and whether they are willing to be randomized. When eligible participants agree to participate, they are invited to come to a location in the municipality of the participant and give their informed consent for participation in the study. Next, participants will be screened on cognition and frailty according to the criteria of Fried et al. and if they have a score of 19 points or higher on the Mini-Mental State Examination and are classified as non- or pre-frail, the baseline assessment will be conducted [31].

### Intervention

**Fig. 1:**
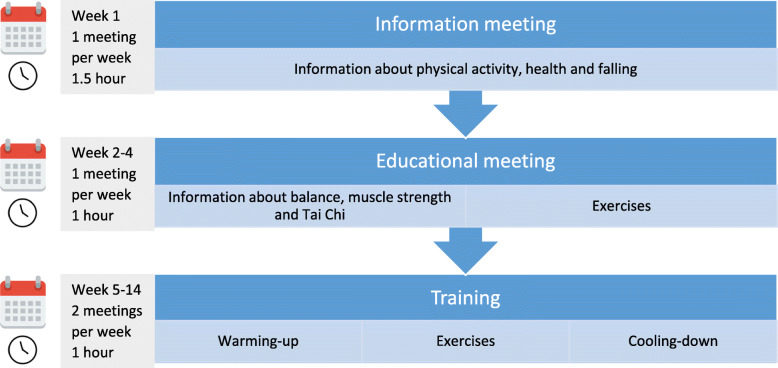
Overview of the In Balance intervention

The In Balance intervention is a fourteen-week group programme for older adults at risk of falls and will be provided by registered and certified physical therapists and exercise therapists [36]. The aim of the intervention is to reduce falls by increasing awareness, balance and strength by combining educational and exercise components. The intervention consists of three phases. The first phase (week 1) comprises one information meeting about physical activity, the impact a fall can have on a person’s life and health, and the purpose of the In Balance intervention. In the second phase (week 2–4), there are three weekly educational meetings about increasing awareness of one’s fall risk and balance disturbance, increasing knowledge about how to implement effective fall prevention methods and getting acquainted with the upcoming training weeks. The third phase (week 5–14) consists of a physical exercise programme with two one-hour training sessions per week. Exercises are derived from principles of Tai Chi, with balance and strength elements and with emphasis on standing strong and shifting weight. Education and Tai Chi exercises are known to be effective in reducing the incidence of falls [37–39]. After each session, the participants receive homework, consisting of conducting exercises learned during the training sessions and reading parts of the textbook belonging to the In Balance intervention. Participants are expected to spend about one hour per week on this homework, distributed over several days. The 3 phases of the intervention are summarized in Fig. 1.

### Control group

The control group will receive written general physical activity recommendations in the form of a flyer. This flyer contains advice on physical activity levels, strength and balance for older adults. For example, a minimal amount of 150 min of moderate to vigorous physical activity per week is recommended, distributed over several days. Also muscle and bone strengthening activities such as walking stairs and balance exercises at least twice per week are advised. Moreover, the health benefits associated with physical activity are explained. These recommendations follow the Dutch Guidelines for Physical Activity [40]. To minimize study attrition, participants of both the intervention and control group will receive regular newsletters during the study and a personal advise on (changes in) physical performance after ending of the study.

### Data collection and outcome measures

#### Primary outcome measure

The primary outcome in this study is the number of falls, with or without injuries, assessed with fall diaries and monthly follow-up telephone calls, including questions on the causes, circumstances and consequences of falls. This combination of pro- and retrospective data collection follows guidelines for conducting fall prevention trials [41].

#### Secondary outcome measures

Besides the number of falls and fall-related injuries, positive effects on physical functioning, physical activity, quality of life and other self-reported outcome measures are measured [42]. The secondary outcome measures will be assessed at three time points during the study period; at entry of the study (baseline, M0), after 4 months to determine the short-term effects (M4) and after 12 months to determine the long-term effects (M12). Societal costs will be assessed at M4, after 8 months (M8) and M12. All secondary outcomes are listed in Table 2 and are described in detail below.

**Table 2:**
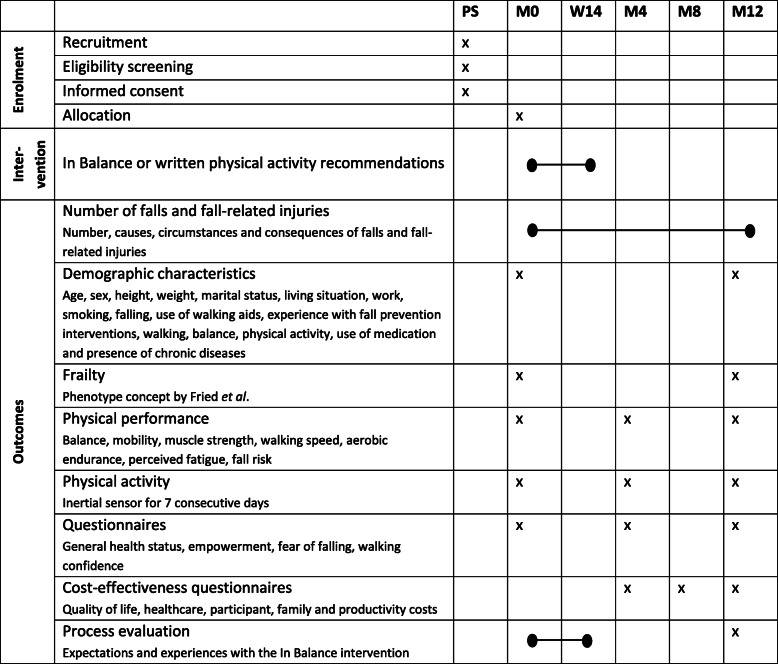
Overview of enrolment, intervention, outcome measurements and time of assessments

PS: prior to study, M0: baseline measurement (pre-intervention), W14: 14 weeks after start of the intervention, M4: 4 months after start of the intervention, M8: 8 months after start of the intervention, M12: 12 months after start of the intervention

#### Questionnaires

The participants will receive and fill in questionnaires at their homes. Participants can choose whether they want to receive the questionnaires online (by email) or on paper.

##### Participant characteristics

Demographic characteristics will be determined at M0 and M12, including age, sex, height, weight, marital status, living situation, work, smoking, fall history, use of walking aids, experience with fall prevention interventions, walking, balance, physical activity, use of medication and presence of chronic diseases.

##### Well-being and fall-related concern

At M0, M4 and M12, several questionnaires will be used. General health status will be determined with the 36-Item Short Form Health Survey [34] and the Positive Health Inventory Tool [43]. Empowerment will be determined with the Pearlin Mastery Scale [44] and the General Self-Efficacy Scale [45]. Concern about falling will be determined with the Falls Efficacy Scale International [46]. Fall risk will be measured with the Longitudinal Aging Study Amsterdam Fall Risk Questionnaire [47]. Walking confidence will be determined with the Modified Gait Efficacy Scale [48]. General quality of life will be assessed using the five-level version of the EuroQol questionnaire (EQ-5D-5L) [49]. Last, to measure quality of life from a broader perspective than health alone, the Adult Social Care Outcomes Toolkit will be used [50].

##### Societal costs

For the economic evaluation, societal costs will be assessed using three retrospective 4-month cost questionnaires at M4, M8 and M12. Healthcare, participant and family costs will be measured using the iMTA Medical Cost Questionnaire [51]. Productivity costs related to paid and unpaid work will be assessed using the iMTA Productivity Cost Questionnaire [52].

#### Physical measurements

##### Physical performance

Physical performance will be assessed at a location in the municipality of the participant on the following time points: M0, M4 and M12. To ensure data quality, assessors, who are blinded to group allocation, will be trained to perform the measurements. The following assessments are included:

1. *Balance* will be measured with the Performance-Oriented Mobility Assessment – Balance, the Four Stage Balance Test and the Timed Up and Go Test. First, the Performance-Oriented Mobility Assessment – Balance evaluates balance during sitting, standing up, standing, eyes closed, rotating and sitting down [53]. Second, the Four Stage Balance Test assesses the ability to stand with the feet together in the side-by-side, semi-tandem, tandem and standing on one foot positions [54]. Third, the Timed Up and Go Test measures the time it takes to stand up from a chair, to walk 3 m, turn around, walk 3 m back, turn around and sit in the chair [55].
2. *Mobility* will be measured with the Performance-Oriented Mobility Assessment – Mobility. This questionnaire evaluates mobility on start of gait, stride length, stride height, stride symmetry, stride continuity, aberrant gait, trunk and foot distance [53].
3. *Muscle Strength* will be measured with the Hand Grip Strength Test and the Timed Chair Stand Test. Hand Grip Strength Grip strength will be assessed using a handheld dynamometer and is calculated as the average of three measurements of the score in kilogram of the dominant hand [33]. Second, the Timed Chair Stand Test measures the time it takes to stand up and sit down from a chair five times as fast as possible without using the arms [56].
4. *Walking Speed* will be measured with the 10 Meter Walk Test [57]. The participants start from rest and walk 10 m at a self-chosen comfortable pace. The score is calculated as the mean time in seconds of 2 attempts.
5. *Aerobic Endurance* will be measured with the 2-min Step Test [58]. The height of the iliac crest and patella is measured and this is marked on the wall. Then tape is placed on the wall half the distance between the two. The participant steps in place, raising each knee to the tape on the wall for as many times as possible in the 2 min period. The number of times the right knee reaches the required height is counted.
6. *Perceived Fatigue* will be scored directly before and after each of the 3 physical performance tests (Timed Chair Stands Test, 10 Meter Walk Test and 2-min Step Test) with the modified Borg’s CR10 scale [59].

##### Physical activity

Physical activity will be assessed with an inertial sensor (DynaPort MoveMonitor Plus, McRoberts BV, The Netherlands) at M0, M4 and M12 [60, 61]. Participants will receive the sensor during the assessment visits at M0, M4 and M12 including corresponding instructions. The sensor will be worn on the lower back for seven consecutive days, preferably day and night, except during water activities, and will afterwards be returned by mail. The sensor includes a tri-axial gyroscope and registers trunk accelerations in vertical, mediolateral and anteroposterior directions, with a sample rate of 100 samples per second and a range of -8 g to + 8 g for a continuous duration of 7 consecutive days. The wearing time of the sensor, the average daily time being upright (i.e. standing and shuffling), in locomotion (i.e. walking, stair walking, cycling) and time spent in sedentary behaviour (i.e. sitting and lying) will be determined. In addition to the amount of daily physical activity, the quality of daily life gait will be calculated from the accelerometry data, as a composite score of characteristics of daily life gait episodes has been shown to be predictive of falls [61]. From the one-week accelerometry data, all locomotion episodes that last 10 s or longer will be selected and divided into epochs of 10 s; gait quality characteristics will be calculated for each of these 10 s epochs. Subsequently, gait quality characteristics will be estimated as median values over the week and the gait quality composite score will be calculated, based on a weighted sum of autocorrelation at stride frequency, power at step frequency, root mean square of the accelerations and index of harmonicity [62].

#### Process evaluation

To evaluate the In Balance intervention, participants of the intervention group will be asked to fill in a questionnaire about their expectations of the In Balance intervention at M0 and their experiences with the In Balance intervention directly after ending the intervention and at M12. Also, the experiences of the In Balance therapists will be assessed with a questionnaire after ending the In Balance intervention. To determine adherence, In Balance therapists maintain an attendance list in which the presence of the participants is logged during the In Balance intervention meetings. Participants will also be asked for the frequency and duration they conducted their homework.

### Data management

Every participant will receive a computer-generated unique identification code at baseline. All data will be collected pseudonymised using this identification code. In the data management system, test results from baseline and post intervention assessments will be collected as well as the documentation on each training session, and for each identification code a digital logbook will be kept in the data management system. To improve data quality, a trained project assistant will verify the data entries and check the case report forms in the data management system after all trainings and assessments are finished. If data are missing, the assistant will check the logbook.

Inertial sensor data will be uploaded to the server of the manufacturer of the accelerometers (McRoberts B.V.) for processing of activity classification of physical activity (walking, stairs walking, standing up, shuffling, cycling) and sedentary behaviour (lying, sitting). This classification will be downloaded from the manufacturer’s server and will be saved together with the raw data on a password protected external hard disk.

### Sample size

To obtain a reduction of 50% in the number of falls between the intervention and control group, a minimum of 106 persons are required per group, at a power of 0.80, beta of 0.02 and alpha of 0.05. Taking into account a dropout rate of 20%, the total required sample size is 256 participants. Hence, we expect that about 16 In Balance intervention groups of 8 participants each, are needed to include the required sample size of 128 participants in both the intervention and control group.

### Statistical analysis

Data will be analysed using SPSS (SPSS Inc., Chicago, IL, USA), RStudio (Version 1.3.1073) and MATLAB (version R2021a; MathWorks Inc., Natick, MA, USA). All analyses will be performed according to the intention-to-treat principle. Numbers and reasons for drop-out and for missing data on the primary outcome will be provided.

#### Demographic characteristics

Data at baseline and post-intervention will be described using means and standard deviations for normally distributed continuous variables, medians and interquartile ranges for non-normally distributed continuous variables, and numbers and percentages for non-continuous variables.

#### Primary and secondary outcomes

The effectiveness of the In Balance intervention in comparison with a control group will be analysed with multilevel mixed model regression analyses for both primary and secondary outcomes. Three hierarchical levels will be included in the mixed models; therapist, participant and time. If necessary, analyses will be adjusted for confounders and stratified for the presence of potential effect modifiers. The primary effect in these analyses is described by the coefficient of the time treatment interaction term. To identify possible differences in intervention effects between non-frail and pre-frail respondents, an a priori subgroup analysis will be performed, stratified for frailty level (non- and pre-frail).

#### Economic evaluation

For the economic evaluation, missing cost and effect data will be imputed using multiple imputation according to the algorithm developed by van Buuren et al. [63]. Linear regression analyses will be used to estimate cost and effect differences between the intervention group and the control group while adjusting for confounders if necessary. Bias-corrected accelerated bootstrapping with 5000 replications will be used to estimate statistical uncertainty surrounding the cost and effect differences. Incremental cost-effectiveness ratios (ICERs) will be calculated by dividing the difference in costs between the groups by the difference in effects. Bootstrapped cost-effect pairs will be plotted on cost-effectiveness planes to show the uncertainty surrounding the ICER. Cost-effectiveness acceptability curves will be estimated to show the probability that the In Balance intervention is cost-effective compared to control for different willingness-to-pay values (i.e. the amount of money that society is willing to pay per additional unit of effect).

### Adverse events

Adverse events are defined as any undesirable experience occurring to a participant during the study, whether or not considered related to the In Balance intervention or the trial procedure. All adverse events reported spontaneously by the participant or observed by the investigator or staff will be recorded. Occurrence of adverse events will be assessed during the monthly telephone calls and for the intervention group during the In Balance intervention by the therapist.

### Trial status

Enrolment into the study will start on September 1, 2021. We plan to complete the recruitment and data collection at baseline by September 2022, with a 12 months follow-up period on falls incidence and incidence of fall-related injuries until September 2023. This study has been registered with the Netherlands Trial Register on 13 February 2021 (NL9248).

## Discussion

This paper describes the design of a (cost-)effectiveness study comparing an implemented group-intervention to prevent falls, In Balance, with general written physical activity recommendations. Exercise programmes have been proven to be effective in preventing falls. In particular, interventions containing challenging balance and functional elements result in the most beneficial outcomes [64–66]. The In Balance intervention includes both balance and physical exercises, and is already proven to be effective in reducing the number of falls among a frail, residential population [24]. Since the target population and duration of the In Balance intervention programme intervention have been adjusted after broader implementation, re-evaluation of the (cost-)effectiveness of the current In Balance intervention is urgently needed.

One strength of this study is that we re-evaluate the widely implemented In Balance fall prevention intervention. In this evaluation, we will stratify for frailty status, which allows us to investigate the impact of the In Balance intervention for different target groups on which the intervention has been extended since implementation. Another strength is that, besides investigating the effectiveness on falls and fall-related injuries, we also study the cost-effectiveness of the In Balance intervention. Little research has been conducted examining the cost-effectiveness of fall prevention interventions in general and no research has been done before on the cost-effectiveness of the In Balance intervention specifically [26]. The cost-effectiveness analysis will indicate whether the added health benefits of the In Balance intervention outweigh the costs.

An additional strength of this study is that we do not only take falls and fall-related injuries into account, which have a large impact on the individual and healthcare, but we will also investigate multiple secondary outcomes such as physical functioning, physical activity, quality of life and other self-reported outcome measures [18]. Despite the health benefits of physical activity, the paradoxical increase in exposure to fall risk should also be taken into account when evaluating falls prevention interventions [67]. Although this study is not powered for the secondary outcome measures, analysis of these outcomes will nevertheless provide insights that are useful to gain more knowledge about the underlying mechanisms of the In Balance intervention on a potential reduction of falls and fall-related injuries. For example, physical activity improves balance control and muscle and bone strength and thus is expected to decrease the number of falls and fall-related injuries [68–71].

This study also includes a process evaluation, to collect information that will be relevant for further implementation of the intervention. Not part of this protocol, but additionally to the process evaluation, we consider focus groups with participants of the intervention group, therapists and other stakeholders to obtain facilitating and hindering factors for implementation. These factors can be taken into account when implementing the outcomes of the study described in this research protocol.

Several challenges of this study need to be mentioned as well. First, the inclusion of 256 participants in this trial, both non-frail and pre-frail, will be challenging. For recruitment, we will involve therapists and their local network over diverse neighbourhoods. Due to the COVID-19 situation, the inclusion of sufficient participants will be extra challenging. We expect that participants may be reticent when considering participating in the In Balance intervention, although the majority of our target population is expected to be vaccinated at the start of our study. Moreover, the social component of the In Balance intervention can be a facilitating factor to participate in this study. In a recent inventory among several In Balance therapists on conducting the In Balance intervention in times of COVID-19, therapists indicated that the In Balance intervention can be conducted while taking the COVID-19 measures into account.

Drop out during follow-up is considered as a threat in this study, particularly in controls due to a lack of attention. To minimize attrition in both the intervention and control group, participants will be closely involved in the study by receiving regular newsletters and a personal advise on (changes in) physical performance after ending the study. In addition, we expect that older adults will appreciate the personal contact during the measurement moments and monthly telephone calls. This personal contact keeps participants involved in the study and lowers the risk of drop out [72]. Another challenge is that even though In Balance therapists will be trained intensively to tailor the level of physical exercises to each individual participant, there will be variance in the training progression of participants in this trial. By training the In Balance therapists and by developing and using standard operating procedures, this variance will be limited. Moreover, this variance represents clinical practice and allows individualized training.

This study includes a considerable number of outcome measures. To limit the burden for participants as much as possible, they will receive questionnaires in their own environment so that they can fill them in in their own time. The physical outcome measures will be assessed at a location in the municipality of the participant and will take about one hour. With the collection of data on the number of falls, we depend on the memory of the participants, with the risk of recall bias. However, we will use an approach consisting of monthly telephone calls in addition to a fall diary, which is considered the most optimal approach to minimize recall bias when reporting the number of falls and fall-related injuries [41]. By personal calls, we will be able to collect detailed information on the causes, circumstances and consequences of falls.

In summary, this trial will provide insight into the clinical and societal impact of an implemented and adjusted Dutch fall prevention intervention and thus has major benefits for older adults, society and health insurance companies. If the In Balance intervention is effective in reducing the number of falls, there are major potential benefits to older adults and the community. Preventing falls could reduce adverse health outcomes, such as disability, hospitalisation and the associated costs [3, 8–11, 16]. Enhanced mobility is expected to improve functioning and result in higher quality of life [8–11]. If cost-effectiveness can be shown, implementation of this intervention will result in more efficient utilisation of health services. Moreover, health insurance companies can make an informed decision on whether the intervention becomes or stays part of insured healthcare. Thus, results of this study will inform healthcare professionals, investigators, and policy makers about timely and (cost-)effective prevention of falls in older adults.

## Data Availability

Only the investigator and study coordinator will have access to the pseudonymised final full trial dataset. A minimal dataset that allows to interpret, replicate and build upon the findings will be available from the corresponding author on reasonable request.
